# Co-producing theory of change to operationalize integrated landscape approaches

**DOI:** 10.1007/s11625-022-01190-3

**Published:** 2022-09-12

**Authors:** James Reed, Colas Chervier, Joli Rumi Borah, Davison Gumbo, Kaala B. Moombe, Teddy M. Mbanga, Alida O’Connor, Freddie Siangulube, Malaika Yanou, Terry Sunderland

**Affiliations:** 1 Center for International Forestry Research, Bogor, Indonesia; 2 School of International Development, University of East Anglia, Norwich Research Park, UK; 3 Centre de Coopération International en Recherche Agronomique pour le développement (CIRAD), Montpellier, France; 4 Faculty of Forestry, University of British Columbia, Vancouver, Canada; 5 Center for International Forestry Research, Lusaka, Zambia; 6 Department of Geography and Environmental Studies, University of Zambia, Lusaka, Zambia; 7 Centre for Social Science Research (CSSR), University of Amsterdam, Amsterdam, The Netherlands

**Keywords:** Environmental governance, Conservation and development trade-offs, Natural resource management, Knowledge co-production, Sustainability science, Transdisciplinarity

## Abstract

Integrated landscape approaches that engage diverse stakeholder groups in landscape governance are increasingly promoted to address linked social–ecological challenges in tropical landscapes. Recent research suggests that a transdisciplinary approach to landscape management can help identify common research needs, enhance knowledge co-production, guide evidence-based policy development, and harmonize cross-sectorial integration. Meanwhile, guiding principles for landscape approaches suggest that identifying common concerns and negotiating a process of change are fundamental to implementation and evaluation efforts. As such, the use of decision support tools such as theory of change models that build ordered sequences of actions towards a desired, and agreed, future state are increasingly advocated. However, the application of the theory of change concept to integrated landscape approaches is limited thus far, particularly within the scientific literature. Here, we address this gap by applying the principles of landscape approaches and knowledge co-production to co-produce a theory of change to address current unsustainable landscape management and associated conflicts in the Kalomo Hills Local Forest Reserve No. P.13 (KFR13) of Zambia. The participatory process engaged a diverse range of stakeholders including village head people, local and international researchers, district councillors, and civil society representatives amongst others. Several pathways, actions, and interventions were developed around the themes of deforestation, biodiversity and wildlife conservation, socio-economic development, access rights, and law enforcement. To make the theory of change actionable, participants identified a need for enhanced cross-sector and multi-level communication, capacity development, and improved governance, while a lack of commitment towards coordinated knowledge exchange and access to information along with poor policy formulation and weak enforcement of rules were among potential impediments to action. Use of theory of change can both inform evidence-based policy design (by revealing place-based challenges and proposing solutions) and support policy mechanisms that promote integration between state and non-state actors (by clarifying actor rights, roles and responsibilities). Co-developing a theory of change for integrated landscape management is inherently context specific, but the process and outcomes of this study should hold relevance across a range of contexts faced with sustainability challenges related to reconciling both conservation and development objectives.

## Introduction

Persistent global challenges of poverty, food insecurity, climate change, and biodiversity loss demand have increased engagement of previously distinct sectors and stakeholders (Stafford-smith et al. 2017). It is now well recognized that traditionally sectorial approaches are inadequately meeting these linked social–ecological challenges and such approaches involve trade-offs and unintended externalities (McShane et al. 2011; Tscharntke et al. 2012; Díaz et al. 2019). Put simply, while sectors of agriculture, forestry, energy, mining, and other commodity supply chains have acted in isolation, the consequences of their actions have had far-ranging impacts that affect other sectors and stakeholders. Tropical landscapes in the global south that are undergoing rapid change exemplify this intersection of seemingly conflicting socio-economic and environmental objectives (Barlow et al. 2018). As such, integrated approaches are increasingly promoted as more holistic strategies to address land-use challenges and natural resource management at a landscape scale (Estrada-Carmona et al. 2014; Milder et al. 2014; Carmenta et al. 2020). Despite lacking universal definition (Scherr et al. 2013; Erbaugh and Agrawal 2017), integrated landscape approaches can be thought of as processes that seek to engage a diverse range of stakeholders with a shared interest in the use and management of a particular landscape, in an attempt to identify the means by which more sustainable and socially just landscape management can be achieved (see for example Sayer et al. 2013; Reed et al. 2016).

Integrated landscape approaches emphasize inter- and trans-disciplinarity, that is, responding to interlinked challenges with enhanced engagement across scientific disciplines and with broader societal actors (Toomey et al. 2015) to bridge research–policy–practice gaps (Sunderland et al., 2009; Rasmussen et al., 2017). This engagement is expected to facilitate collaboratively produced knowledge (Tengö et al., 2014; Norström et al., 2020) that enhances social–ecological system sustainability and multifunctionality (Brandt 2003). As such, advocates of integrated landscape approaches draw from a range of disciplinary fields. For the past twenty years or so, landscape approaches have been advocated for—and adopted by—numerous national and international conservation and development agencies and conventions (Reed et al. 2015a, b) and have now been applied across a range of contexts (Estrada-Carmona et al. 2014; Milder et al. 2014; García-Martín et al. 2016; Reed et al. 2017; Zanzanaini et al. 2017).

However, despite their increasing prominence, actual evaluation of integrated landscape approaches is nascent, and there remains a lack of clarity on how best to ensure that the diversity of needs or objectives is being met. A stated ambition of integrated landscape approaches is to pursue a scenario whereby there are more winners and less losers (Sayer et al. 2014). Of course, this is desirable, but it is also accepted that tropical landscapes are inherently complex, rendering success subjective and therefore very much in the eye of the beholder (Meinig 1979; Tress et al. 2001): an optimal outcome for one stakeholder may not be so for another. Therefore, implementation and particularly evaluation of integrated landscape approaches must pay attention to not only the biophysical landscape impacts, but also the social implications, outcomes, and perceptions of effectiveness and equity. Indeed, the importance of stakeholder perceptions of conservation or development interventions are increasingly recognized as being influential to conservation and development outcomes (Bennett 2016); perceptions influence local buy-in and condition subsequent behavior (Bennett 2016; Carmenta et al. 2017; Abukari and Mwalyosi 2020) and are therefore crucial to the acceptance and effectiveness of landscape approach adoption.

Despite the variation in types of landscape approach, there is some consistency across the literature related to principles of good practice. Preeminent amongst integrated landscape approach design principles are those proposed by Sayer et al. (2013), who advocate for (amongst others) engaging multiple stakeholders to identify common concerns and negotiate a transparent change logic to reconcile land-use objectives, principles that have been subsequently endorsed by multiple scholars (e.g. Freeman et al. 2015; Reed et al. 2016; Ros-Tonen et al. 2018) and to some degree applied in practice. For example, USAID’s major landscape-scale initiative in Indonesia, LESTARI, used the ten principle framework as a guide for implementation (USAID LESTARI 2019) and the same principles have been used to guide and assess landscape-scale initiatives in Uganda (Omoding et al. 2020) and restoration efforts in forested landscapes in Ghana (Acheampong et al. 2020).

Meanwhile, principles developed for co-production of knowledge to address complex contemporary sustainability challenges emphasize the need for context-based, pluralistic, goal-oriented, and iterative processes (Norström et al. 2020). Well-coordinated knowledge co-production processes present an opportunity to integrate academic/non-academic and abstract/context-specific knowledge in an attempt to co-develop responses to sustainability challenges and translate new knowledge into action (Hoffmann et al. 2017; Pohl et al. 2021). The literature on knowledge co-production recommends using methods including scenario building, mapping, and the development and use of boundary objects—of which a theory of change could be considered an example. There are clear overlaps between landscape approaches, knowledge co-production, and theory of change principles and methods. However, to the best of our knowledge, there has yet to be a documented attempt to develop a causal theory of change (Qiu et al. 2018; Chervier et al. 2020) in a collaborative manner applying integrated landscape and knowledge co-production principles. As part of a broader attempt to operationalize integrated landscape approaches, here we attempt to incorporate these principles and draw from the wider literature to co-produce a theory of change to pursue more inclusive, sustainable, and equitable landscape management in the Kalomo District of Southern Zambia.

### Theoretical framework for participatory theory of change

Our use of a participatory approach to co-produce a theory of change for an integrated landscape approach is novel and responds to the lack of a clear and definite methodology. Despite numerous endorsements for theory of change use within the literature on integrated landscape approaches (e.g. Sayer et al. 2013; Freeman et al. 2015; Ros-Tonen et al. 2018), we were unaware of either a definitive methodology for applying theory of change to such approaches, or a detailed account of theory of change being (co)produced within the recent literature related to landscape approaches (Sayer et al. 2016). More typical from our experience has been a tendency for project implementers to *independently* and ex situ develop a theory of change or logical framework. While such pre-conceived, externally produced logframes can be useful for clarifying underlying project assumptions, they can also be misaligned with local realities, lack the necessary flexibility to adapt to unplanned or unforeseen events, and—perhaps most crucially—inadequately incorporate local knowledge and suppress innovation and motivation to engage (Sayer and Wells 2004). Nevertheless, more participatory modes of theory of change (or causal chain) methodology has been developed, applied and documented in related fields, for example in public health, political science, international development, and illegal wildlife trade (Vogel 2012; Biggs et al. 2016; Breuer et al. 2016), while Qiu et al. (2018) proposed a methodology for interdisciplinary causal chain development to link sectors of health, conservation, and development. Moreover, it may well be the case that practitioners of integrated landscape approaches are using the theory of change in their implementation and perhaps even doing so in participatory environments, but have not thus far contributed to the process, implications, or challenges to the scientific literature (Sunderland et al. 2009).

**Fig. 1:**
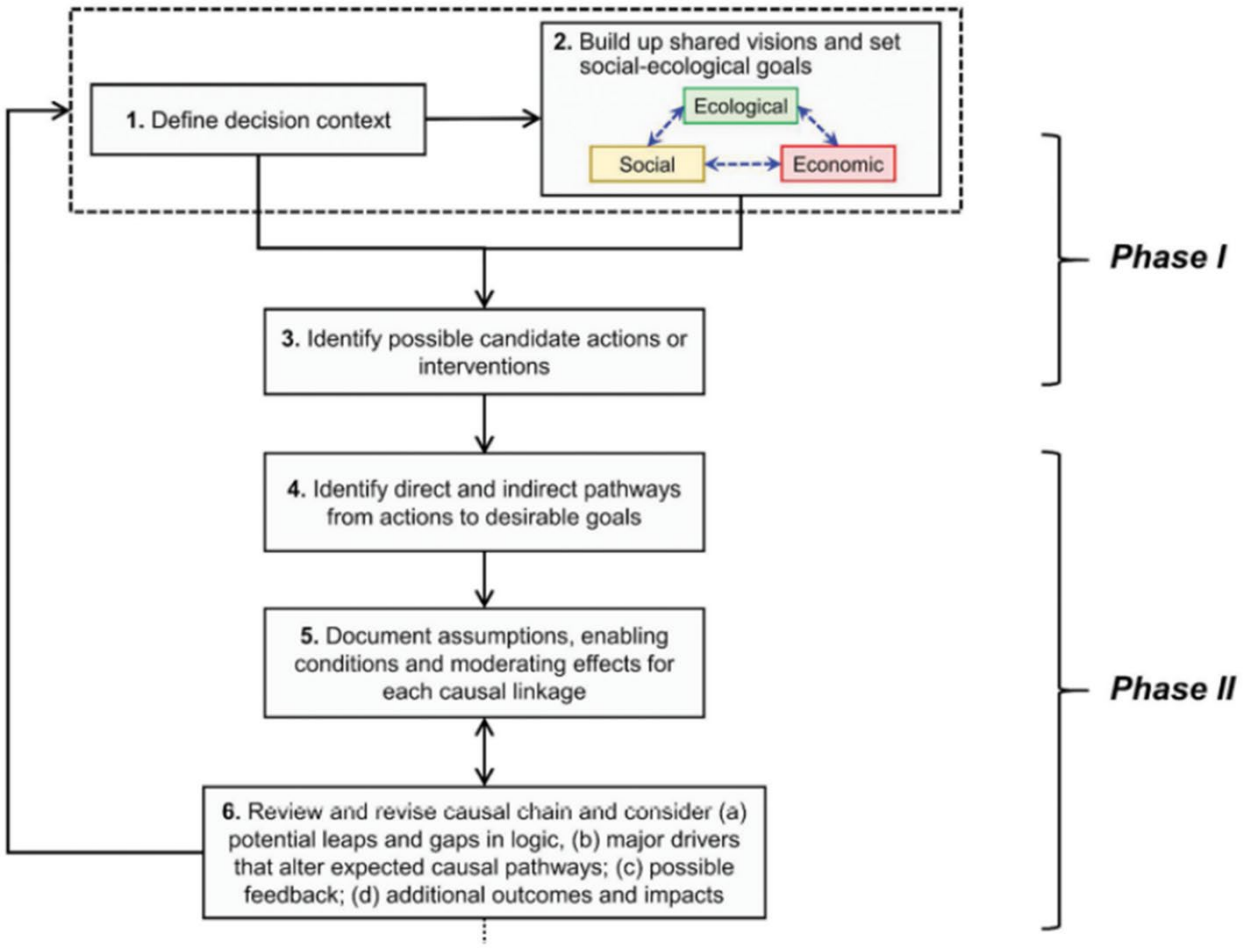
The first two phases of causal chain methodology (source: Qui et al. 2018)

**Fig. 2:**
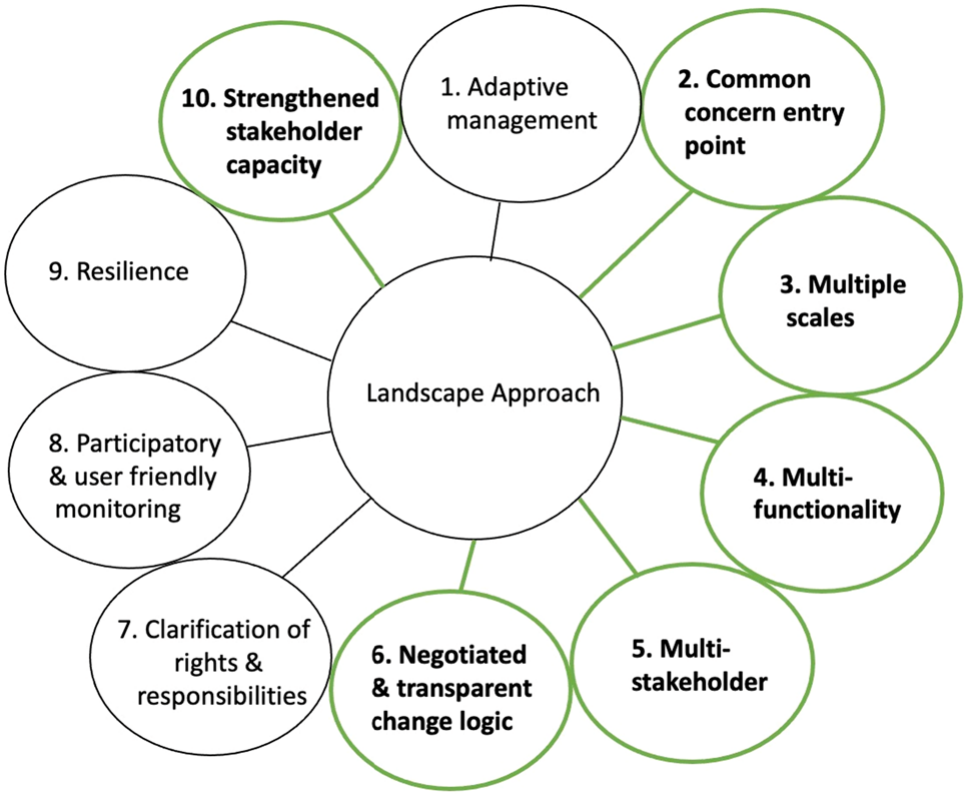
The ten principles for a landscape approach (source: Sayer et al. 2013)

We contribute to filling this gap in the literature by collaboratively developing a theory of change for an integrated landscape approach to address persistent land-use issues in Kalomo. We drew inspiration from the work of Qiu et al. (2018) and Sayer et al. (2016), in particular, focusing on phases one and two of the Qui et al. methodology (see Fig. 1) and paying attention to the need to consider multiple and potentially conflicting stakeholder objectives and epistemological understandings as outlined in Sayer et al. We further supplemented these guidelines with lessons learnt from our own experiences in multi-stakeholder landscape-scale processes and the broader literature on landscape approaches and knowledge co-production, specifically incorporating principles of landscape approaches such as those (2–6 and 10) that emphasize engaging multiple stakeholders from across multiple sectors to establish common concerns and negotiate a change logic that enhances capacity and multifunctionality (Sayer et al. 2013) (see Fig. 2) and principles of knowledge co-production (see “Introduction”) as well as lessons learnt that accentuate issues of relevancy, saliency, and inclusivity (Djenontin and Meadow 2018).

## Methods

### Study site

**Fig. 3:**
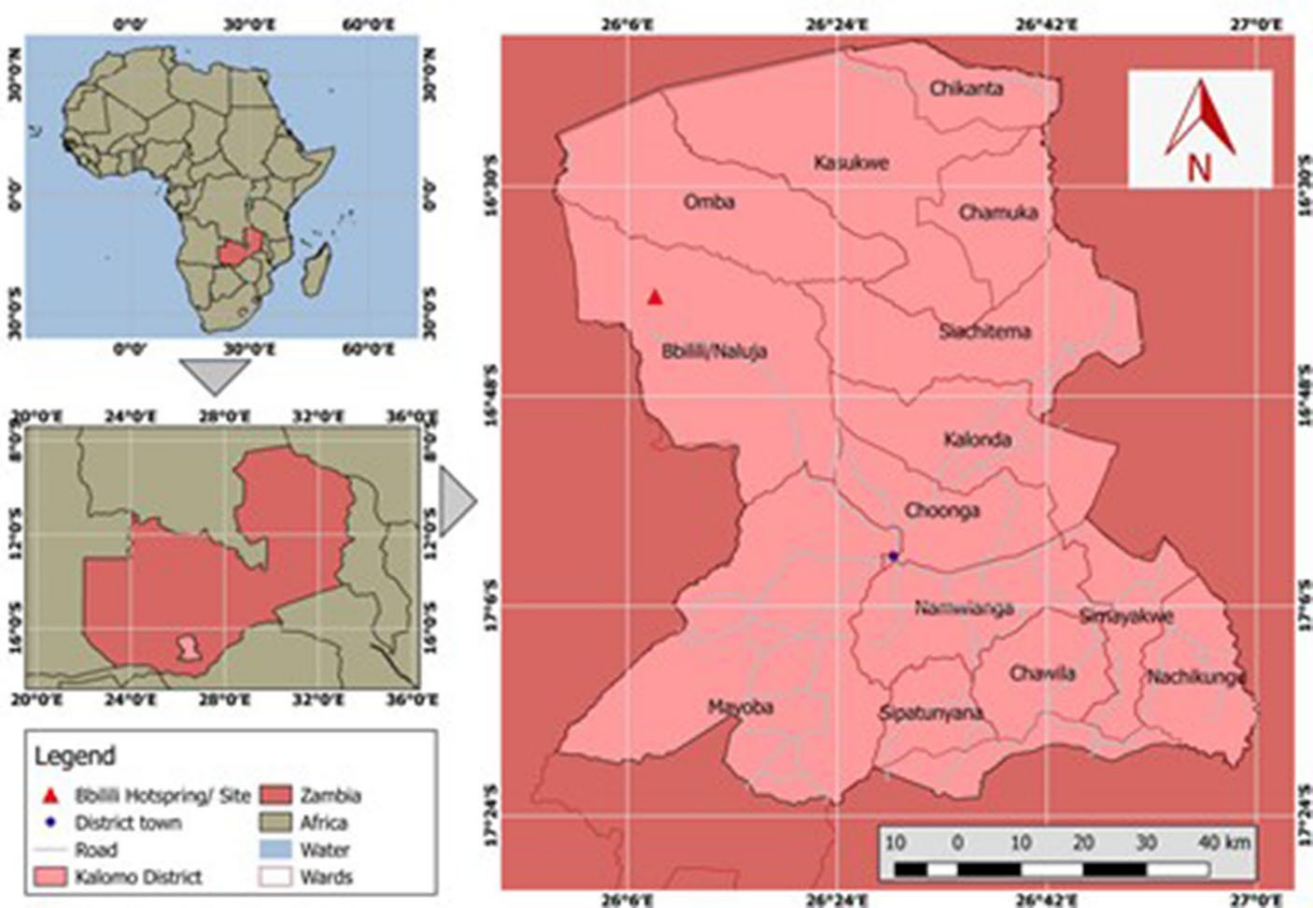
Location and administrative map of Kalomo District

The development of the participatory theory of change was embedded within the COLANDS (Collaborating to Operationalize Landscape Approaches for Nature, Development and Sustainability) initiative that is operationalising integrated landscape approaches in three tropical countries: Indonesia, Ghana, and Zambia (Reed et al. 2020c). It is the latter of these countries that this study was conducted for and in, specifically in the district of Kalomo in Zambia’s Southern Province (Fig. 3). Kalomo is a district encompassing 8075 km^2^ of mixed vegetation cover, predominated by riparian forests with the Miombo, Mopane, and Kalahari woodlands. The local economy is largely agricultural and has been traditionally based on cattle and maize, and lately, tobacco, with more recent expansion of agrarian activities to include goat, pig, sheep, poultry, groundnut, and cotton production, amongst others. The district encompasses the Kalomo Hills Local Forest Reserve No. P.13 (KFR13), a legally protected 1,369km^2^ reserve established in 1970 (Ministry of Land and Natural Resources).

With a growing and more demanding local population with increasing access to national and international markets, but beset by water scarcity and land degradation, Kalomo typifies a contested landscape and has recently been characterized by weak institutional linkages, tensions between scales of governance, and an escalation of disputes over land use and access. These challenges and contestations are further exacerbated by the impacts of climate change, especially prolonged droughts, and a generally uncoordinated approach to addressing local land-use issues (Moombe et al. 2020).

The selection of the study site followed an extensive scoping mission that considered potential sites across the country based on various criteria, including landscape heterogeneity and dynamics, existing stakeholder networks and decision-making fora, drivers of environmental change, potential constraints, enabling conditions, and factors for scalability for integrated landscape approaches (see Moombe et al. 2020 for more detail).

### Stakeholder identification

From our previous research in Zambia and Kalomo District (Moombe et al. 2020) and ongoing research activities^1^ including stakeholder perception and network analysis (forthcoming), we were able to identify a range of revelant stakeholders with an interest in the future management of the landscape. In total, 51 participants were invited representing a broad spectrum including village head people, social, biophysical, and interdisciplinary researchers, government officials, non-governmental officials, traditional chiefs, and private sector representatives. From this invite, a total of 46 participants (nine female) joined the theory of change workshop which was held in Choma, Zambia, from 17 to 19 February 2020. It is significant to note that despite responding positively to invitations, no private sector actors joined the workshop (see “Study limitations” below).

### Building a participatory theory of change

With 46 participants, it was decided to develop three groups to work independently on building theories of change, with plenary sessions at the beginning and end of each day (see more below). Firstly, it was felt that the smaller number of participants per group would make the task more manageable and enhance the potential for more inclusive discussion. Facilitators were on hand to provide support and ensure all participants were able to provide input—these facilitators were researchers (representing CIFOR and Care International) with sufficient and relevant training and experience to be careful not to introduce bias and ensure that all participants were able to contribute irrespective of age, gender, or social status and that discussions were not dominated by any particular individual or group. Secondly, it was felt that by having three groups complete each of the steps towards the theory of change, each group could then critique and provide feedback to the others as the process progressed. The groups were selected randomly ensuring that neither was dominated by any particular stakeholder group. Each group had flipcharts, paper, and marker pens and were encouraged to document each step of the process, including developing their own problem trees and action pathways where appropriate (see steps three and six below for example).

**Fig. 4:**
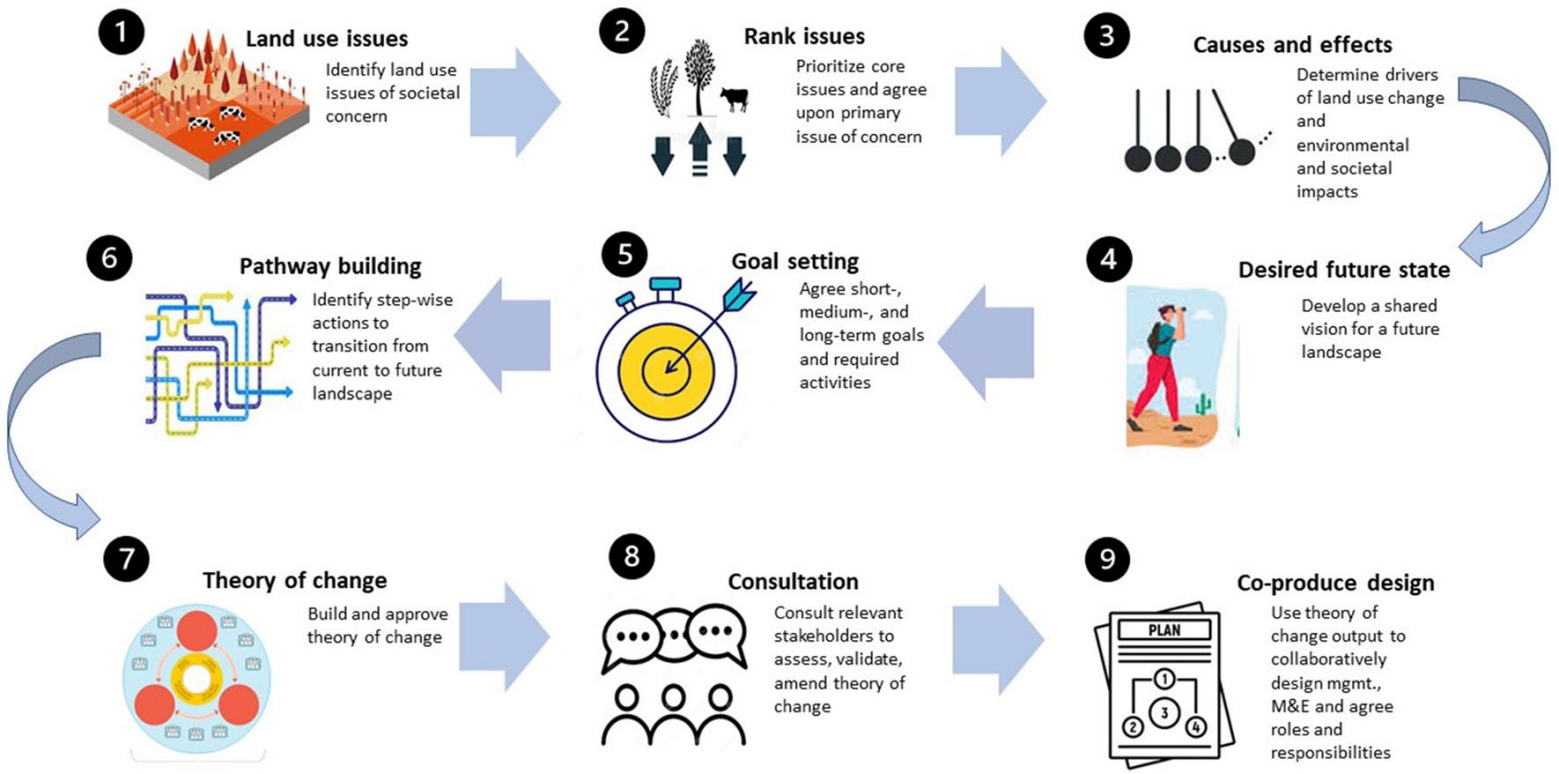
Nine steps of the participatory theory of change development

Prior to selecting the working groups, participants were asked to individually record what they perceived to be the (up to) three most pressing land-use issues in the district (step one). This information was then collated and shared with the groups who would then use it to inform and/or validate their theory of change development. However, being cognizant that theory of change is a largely academic construct, we were keen to avoid the use of overly technical terminology (such as the term “theory of change”) that could impede progress. We therefore broke the process down into five further sequential steps to be conducted within the workshop setting and then three additional steps to be conducted upon conclusion of the workshop (Fig. 4).

Once the groups were randomly selected, the second step was to use the aforementioned collated land-use issues as a basis to discuss the current state of the landscape in Kalomo and the associated land-use sectors and practices operational in the district. Participants then aimed to identify the current core land-use issues and challenges perceived to be inimical to achieving sustainable and integrated landscape management—working in groups, participants used the information generated in step one as a basis to establish and achieve consensus on the main issues. Once a complete list was identified, these were then classified within broad themes, loosely prioritized with agreement on the primary land-use issue in Kalomo, i.e. identifying a common concern (Sayer et al. 2013; Ros-Tonen et al. 2018).

For step three, participants used problem tree analysis (Narayanasamy 2009) to specify the causes and consequences of the main issue identified in step two—this step enabled the identification of the direct and indirect drivers of land-use change as well as the resulting environmental and societal impacts. The output produced from this step (i.e. the problem tree, see Fig. 6 in “Results” below) was then used to inform the following steps four, five, and six. Step four required participants to develop a ‘desired future state’—essentially how the group envisages the landscape in a distant idealized future (see Table 1 in Results). For this step, participants were encouraged to think about temporality, but providing a specific end date was not a necessity (alluding to the ethos of integrated landscape approaches as iterative processes rather than specific targets).

In step five, participants developed sets of short, medium, and long-term activities and goals that would be necessary to implement and achieve to transition towards the previously identified desired future state. For this step, participants were encouraged to allocate these activities and goals to appropriate, logical time frames (i.e., short term could be within the next 3–5 years). The next step (step six) required participants to draw on the information they had generated in the previous steps to then co-construct the pathways that depict a transition from the current landscape state to the desired future state. These pathways should represent an ordered and logical sequence of actions (avoiding any ‘leaps of faith’), identifying the necessary interventions or policy options to move between steps, and consider potential social–ecological feedbacks (Qiu et al. 2018).

On completion of each of steps two, three, and six, the groups re-convened to present their progress to each other and provide feedback. Once the theory of change pathways was complete, it was decided that a small group of researchers would then synthesize the three outputs to produce a single theory of change to be shared with the whole group for input and ultimately approval (step seven). The approved theory of change would then be distributed to identified stakeholders who were unable or unwilling to join the initial workshop but have an interest in—or will be impacted by—the future management of the Kalomo landscape. These stakeholders would be asked to either validate or appropriately amend the theory of change (step eight). The final theory of change is then used as a basis for designing a landscape monitoring and evaluation framework, and as a guide for a co-produced future action plan with individual and institutional roles and responsibilities clearly identified and agreed upon (step nine). Such an action plan should specify the need to periodically revisit the theory of change and re-evaluate goals and activities as appropriate.

## Results

For the “Results” (except where stated), we present the synthesized findings of the working groups.

### Land-use issues in Kalomo District

**Fig. 5:**
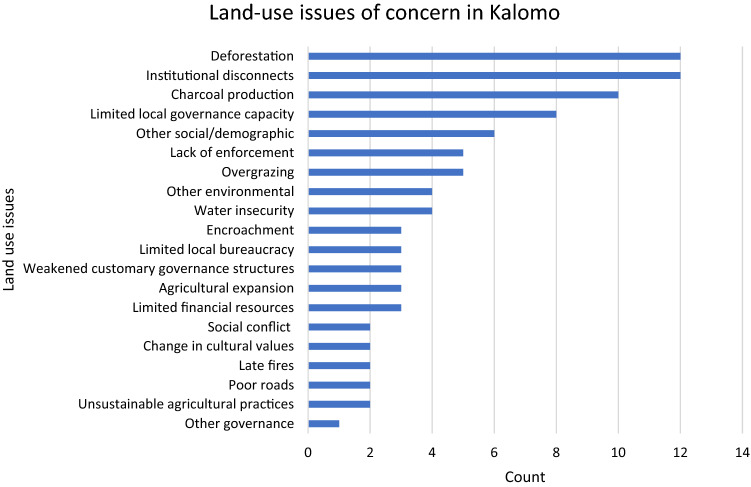
Most pressing land-use issues in Kalomo, as identified by workshop participants individually selecting up to three most pressing issues of concern

From the preliminary activity that required participants to individually identify up to three current land-use issues each, there were collectively 92 elected issues representing a diverse range including a growing population, erosion, weak law enforcement, fire management, and water supply amongst many others. The most commonly perceived land-use issues related to deforestation, weak institutional linkages, charcoal production, and governance concerns (see Fig. 5).

**Fig. 6:**
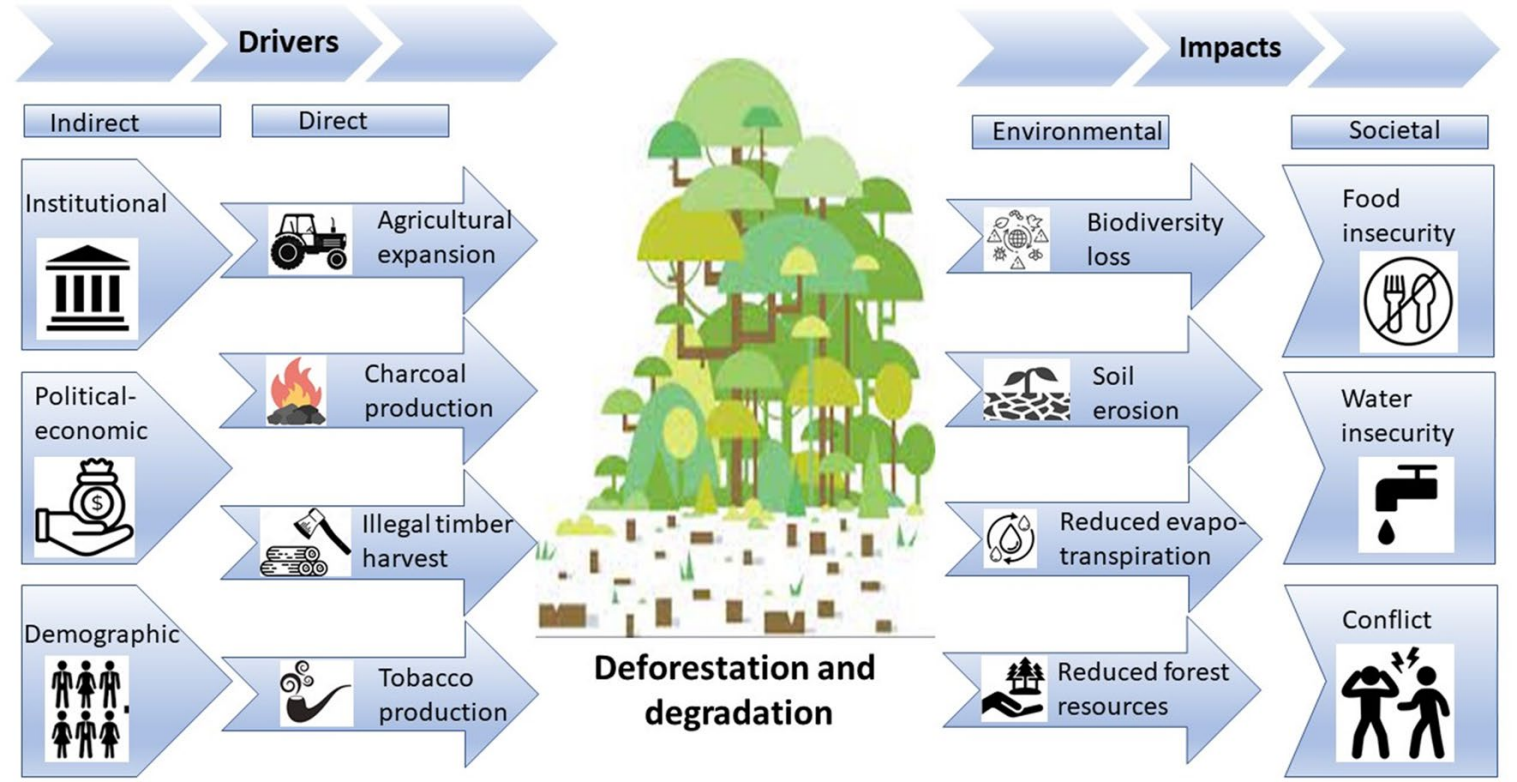
The core land-use issues of concern and the primary land-use change drivers and impacts in Kalomo as identified through the participatory use of problem tree analysis

It was acknowledged that while deforestation and degradation in the Kalomo Hills Forest Reserve were the core issues, there were a range of other inter-related land-use issues, many of which represent direct and indirect drivers of forest loss. In turn, ongoing deforestation and degradation is generating both environmental and societal impacts in the region. We have categorized these drivers and impacts below (see Fig. 6) and briefly elaborate based on conversations within the workshop.

## Direct drivers of deforestation and degradation

The main economic activity in the landscape is agriculture and as such *agricultural expansion* is the primary driver of deforestation and degradation in the KFR. Settlers open spaces to produce maize and other crops and establish pastures. Maize is the dominant crop under cultivation, followed by tobacco cultivation which has seen recent expansion. Aside from maize and tobacco, other crops grown include beans, cowpeas, sunflower, groundnuts, and sweet potatoes, with the growing of the latter increasing in prominence due to enhanced market access in the town of Livingstone and some neighbouring countries such as Botswana and South Africa.

In the Southern Province, *charcoal production* and trade started in the late 1990s, became established in the 2000s and has become increasingly widespread since. Charcoal is produced from both customary and state lands including within the KFR. Finally, the *illegal harvest of timber* is another increasing driver, as population growth within the KFR has increased demand for fuelwood and pole wood, while inhabitants also provide timber to furniture manufacturers as an additional source of income.

## Indirect drivers of deforestation and degradation

A reasonably long list of *political–economic and institutional issues* and constraints were identified by stakeholders, and the source of these issues ranged from local to national scale. The perceived importance of the issue was also variable depending on the individual, with some attributing greater significance to certain issues. For example, the issue of clarifying and enforcing chiefdom boundaries is a primary issue for traditional chiefs, but perhaps of lesser importance for government officials or researchers who, respectively, suggested that resources could be better directed elsewhere and that established and enforced boundaries do not necessarily ensure compliance. Nevertheless, there was greater consensus on other issues that contribute towards ineffective or inadequate landscape governance. A significant, and commonly shared, concern is a sense of weak institutional linkages and a general lack of coordination amongst actors and entities invested in the landscape. This institutional failure is perpetuated by unclear or outdated policy formulation processes as well as overlaps and conflicts between current laws and policies in place—particularly between statutory and customary institutions. This is further problematized by unclear decision-making and enforcement authority and leads to significant or contested power relations, marginalization of certain actors or groups and inequitable access to resources, and poor dissemination of policy guidance, knowledge, and examples of good practice.

Deforestation and degradation are further indirectly driven by a range of *demographic factors*. These include a growing local population as well as increasing in-migration, which has resulted in an increased demand for natural resources. The increasing population has also led to several issues and conflicts related to disputed land boundaries. For example, the initial communal ownership of natural resources led to a situation in which their management was tasked to traditional leadership, but was inadequately undertaken, further generating land-use and access disputes (interview with Chief Siachitema).

## Environmental impacts of deforestation and degradation

The *loss of biodiversity* includes losses to both fauna and flora. The loss of forest in the reserve has had a negative impact on floristic biodiversity with some plant species and tubers now in short supply. The *Munkoyo* tuber (*Rhynchosia heterophylla*) has been severely depleted due to overharvesting for sale and the establishment of crop fields. This has had a negative impact on the local diet, which depends heavily on the use of *Munkoyo* for making a traditional brew known as *Chibwantu*. Due to human habitation, various wildlife species have migrated westwards away from the forest reserve. Several wildlife species such as antelope, waterbuck, lechwe, wildebeest, zebra, buffalo, elephant, bush pig, and porcupine among others were a common sight up to the late 1990s. As human habitation increased, the number of animal sightings has significantly reduced.

Several streams, including Sichikwalula, Nazibula, and Simwaanda, within KFR provided a source of water for wildlife and supported a variety of fish species, an important protein-source in the local diet as well as as a source of income. However, disturbances to the waterways resulting from several activities, including deforestation, have led to the depletion of fish in most of these streams. Exacerbated by reduced water in these water bodies, animal migration became inevitable thereby compromising the diversity within the landscape.

The soils in KFR, classified as plateau soils, are generally poor for crop cultivation due to low nutrient content and poor moisture-retention capacity. Deforestation and unsustainable agricultural practices have led to further *soil erosion* with soil fertility declining over time as population has risen along with increased demands—and limited supply—for *forest resources*, increased monocropping with overcropping, and concomitant increased use of synthetic fertilizers. This combination of decreasing forest cover and increasing (unsustainable) crop production also negatively impacts *evapotranspiration* with potential subsequent reductions in rainfall and increased drought events.

## Societal impacts of deforestation and degradation

Environmental factors such as reduced soil fertility, increased soil erosion, and loss of biodiversity alongside climatic factors all serve to negatively impact crop production and therefore increase local *food (and water) insecurity*. With farmers reliant on rains for crop production, the increase in drought events has led to volatility in maize yields, while excess use of synthetic fertilizer has eroded soil quality and consequently led to further agricultural expansion. Meanwhile, forest loss due to expansion of agricultural fields and settlements has led to the depletion of edible forest resources such as wild fruits, tubers, and leafy vegetables that not only act as a food source, but also seasonally as an additional income source.

Siltation of many streams, e.g. Simwanda stream near head person Simboola’s village, has resulted in reduced access and lower quality of water. Waterways that once provided sources of clean water are heavily silted and shallower. Mitigation measures against this challenge include drilling boreholes and creating wells including on streambeds to reach the water beneath a layer of silt. This has created challenges for the local population, especially women and children that are primarily tasked to draw water for domestic use. There is also *increased conflict* around watering points, as claims and counterclaims of ownership are increasingly common. Finally, institutional and administrative shortcomings related to uncertainty over boundaries and access rights to land, trees, and water have further heightened stakeholder contestation.

### Other emerging and cross-cutting threats

In common with other regions in sub-Saharan Africa, the impacts of climate change are increasingly prominent in Southern Zambia. There was partial drought in 2019 that impacted crop and livestock production. Transhumance to the dams at Nangubo and Simwaanda and at Bbilili Hot Springs has usually mitigated the impact of drought in KFR as the dams normally have more water than streams within and nearby the reserve. The loss of livestock, especially cattle used as draft animal is a direct loss in means of production and reduction in the efficiency in crop production. These losses are further intensified as income from sale of livestock in times of need is also compromised. As such, other off-field practices often develop as a consequence of the impact of drought. For example, wood extraction for pole and fuelwood to meet family household needs, with it becoming increasingly common to find charcoal kilns and heaps of firewood owned by the same farmers that practice cropping.

The use of fire poses another increasing threat to the fauna and flora of the landscape. Bush fires typically result from land preparation for cropping, the process of charcoal production, and during the hunting season. Burning tree trunks in preparation for cropping is a common practice in the landscape, particularly amongst elderly men, as it is less labour intensive than manually clearing land. Sometimes this leads to uncontrolled fires that envelop large areas of the landscape. In new settlements such as those along Ngoma Road, at Katondo in Mazwanga Village (Siachitema), large areas of forest have been burnt for settlement and cropping areas. Fire is also used for hunting and several fire scars are prevalent during most parts of the dry season.

### A desired future

Working in groups, participants were tasked with reaching consensus on the landscape characteristics and institutional reforms that they would like to see achieved in the future. Given the already agreed common concern related to deforestation and forest degradation, it was unsurprising that each of the groups placed an emphasis on interventions that would either reduce the current rate of deforestation, promote reforestation, or move towards more sustainable use of natural resources as a priority. The groups recognized a need to deliver training that would improve current farming practices. There was also a strong emphasis on improving the current governance structures in place and securing greater coordination between the various decision-making scales to enable integrated planning and management (Table 1).

**Table 1:** A desired future

Group 1	Group 2	Group 3
Reduced rate of deforestation and forest degradation	Reforestation and afforestation	Sustainable use of natural resources
Improved mgmt. of grazing lands and pastures	Rivers flow again	Harmonized policies e.g. between village acts, chiefdom, and national polices
Enhanced performance of institutions to achieve their mandates	Positive coordination—proper integrated planning	Strong governance systems
Restore and maintain biodiversity	Climate smart and conservation agriculture	People taught on how to sustain the environment
	Laws on conservation of natural resources should be harmonized	Farmers learn conservation agriculture
	Laws properly enforced	Enforcement of environmental laws at all levels
	More consultation between chiefdoms, departments, and communities	Integrated land-use plans
		Exchange of knowledge between chiefdoms

The landscape characteristics and reforms each group hoped to realize in a future Kalomo. The information within the table is presented ad verbatim

In summary, the participants envisioned a future state for Kalomo where improved consultation across scales of influence (particularly between and across chiefdoms, departments, and communities) would enable properly enforced and harmonized laws. They felt this could lead to reduced deforestation, restoration of forests and biodiversity, improved management of grazing lands and pastures, and sustainable use of natural resources. It was considered that such a state would then deliver improved river flow, water and food security, rural infrastructure, income, and livelihood benefits.

### Short-, medium-, and long-term activities and goals

Participants used the information generated from steps three and four to guide the identification of a suite of activities and goals that would facilitate a transition towards more sustainable and inclusive landscape management in Kalomo. These goals and activities were categorized within a range of near to long term (Table 2).

**Table 2:** Short to long-term goals and activities required to facilitate a transition towards more sustainable landscape management in Kalomo (step five) as developed by workshop participants. This table represents a synthesis of the goals and activities proposed by the three groups

Near term (less than 2 years)	Short term (less than 5 years)	Medium term (5–10 years)	Long term (more than 10 years)
More and better-informed stakeholders on natural resource management	Reduced reliance on charcoal production as energy source and alternatives sought	Restored biodiversity	Increased income and human well-being
Improved capacity on grazing land and pasture management	Reduced livestock mortality	Increased number of livestock	Restored and sustainably managed landscape
Increased funding for natural resource management	Improved staffing levels	Reduced land-use conflicts	Improved service delivery
Improved cross-scale communication channels	Increase in extension services	Conservation agriculture more widely understood and practiced	Improved river flow
	Reforestation and afforestation programmes in place	Increased crop production	Enhanced revenue flow from timber and NTFP production
	Greater collaboration between government departments and traditional chiefs	Properly enforced and harmonized laws	Improved wildlife and water conservation measures
	Clarity over chiefdom boundaries and resolution of boundary disputes	Increased soil fertility, reduction in soil erosion	Improved access to healthcare and education
	Greater inclusion of local communities and especially women in natural resource management	Enhanced clarity on landscape management roles and responsibilities	
		Further research on forests and natural resources	

### Theory of change development

**Fig. 7:**
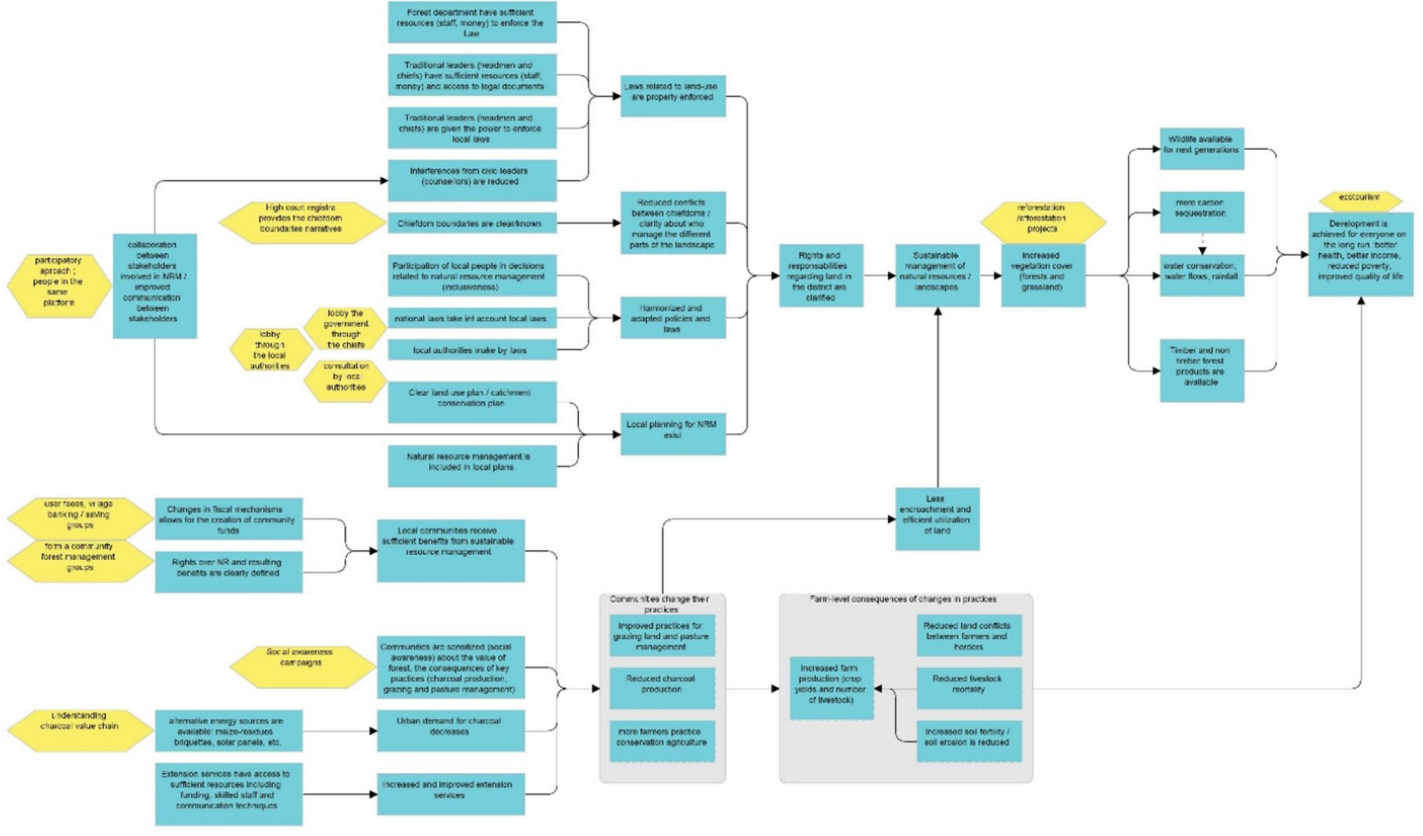
Resulting theory of change model to be circulated for consultation. It is important to note that this theory of change is simply the result of the workshop, is not a management plan, and remains subject to change after further consultation with relevant stakeholders (step eight)

The resulting theory of change model presents the causal pathways to transition from the current (undesirable) landscape dynamics towards the desired future state. This model was developed by the research team with consultation and input from the workshop participants and represents the synthesized outputs of the three group’s pathway building exercise (step six). There are several consistent and cross-cutting themes that are considered fundamental enabling conditions for progress. For example, participants shared a strong consensus for: improved actor and institutional coordination and enhanced collaboration of actors across multiple scales to improve collective decision-making; a clarification of land-use boundaries; better enforcement of regulations; recognized and secured access rights; and enhanced resources for alternative and/or sustainable land-use practice (see Fig. 7). We further elaborate on the implications of these themes in the sections below.

## Discussion

### Perceptions of landscape dynamics

Participant observations on land-use issues and land cover change (steps 1 and 2) align with findings from the recent literature and are consistent with recent concern locally (both from inhabitants and decision-makers) over the governance of the KFR. The forest cover change in the period 1984–2018 in the KFR declined from approximately 138,844 ha in 1984 to 42,409 ha in 2018, representing a loss of 96,435 ha or 2836 ha/two percent of the total cover per annum. In the process, shrubs and grassland have replaced forests in some places (Mbanga et al. 2021). As identified, this rate of forest loss results from a range of direct and indirect drivers, principle amongst which are agricultural expansion, population growth, and unsustainable natural resource extraction.

The population of the Kalomo District is growing at a rate of 4.4% (CSO 2012). Early migration into Kalomo resulted from natural drivers, such as the creation of the Kariba dam, economic factors, and early wars (Nchito 2014). The subsequent population increase was initially due to in-migration into the Kalomo Hills Forest Reserve, but has more recently been due to natural increase. Most recently, migration into the reserve has reduced and there is more emigration due to a perceived loss of productivity of croplands (Mbanga et al. 2021). However, the general increase in population within Kalomo has increased the demand for, and pressure on, land and natural resources. These resources have been stressed due to overexploitation and unsustainable harvesting methods.

Fifty percent of the *maize* produced by Kalomo District (110,000 tons) originates from the forest reserve and its periphery (Moombe et al. 2020). Recent expansion of *tobacco cultivation*, especially the Virginia variety, has been prevalent in Kalomo District, although not across all chiefdoms. For example, in Chikanta chiefdom, a decree prevents growing tobacco due to the associated destruction of trees. The practice of growing tobacco is however widespread in Siachitema chiefdom. The location of the Tobacco Board of Zambia within Siachitema led to active promotion of the cultivation of the crop. There are several outgrower schemes in the landscape that supply tobacco to several tobacco companies. According to Mbanga et al., (2021), Virginia tobacco fetches a higher market price but requires fire curing. Therefore, trees are also lost to support tobacco production.

Unsustainable and informal charcoal production is a persistent challenge in the district. The genesis of the commercial charcoal trade is strongly associated with the climatic changes that affected the agropastoral culture of the Tonga people (Moombe at al. 2020). Urban areas within and beyond Kalomo District provide the market for the commodity. For example, only a small proportion of charcoal produced from KFR is consumed within Kalomo Hills among civil servants at schools and rural health centres (Mbanga et al. 2021). Nevertheless, the production of charcoal, previously less prevalent and used as a contingency practice, has rapidly grown in prominence and is now a major source of household income for many within the KFR.

### Implications for landscape governance

Throughout the workshop, governance failings and challenges were consistently identified and for the pathways identified to be actionable, some governance reforms will be necessary. For example, a perceived lack of access to information tends towards a lack of trust, continued weak enforcement of rules can stifle progress towards shared goals, and lack of coordinated knowledge exchange can further marginalize rural communities. Nevertheless, participants engaged in healthy and constructive discussions and through the course of the workshop and development of the theory of change were able to highlight three key, and related, areas for improvement.

The need for improved *cross-scale dialogue* was well recognized and acknowledged by all participants. This need for greater actor engagement is consistent with recent scholarship on environmental governance generally that promotes multi-level (Newig and Koontz 2014) or polycentric (Nagendra and Ostrom 2012; Mcginnis 2016; Chazdon et al. 2021; Djenontin and Zulu 2021) governance structures as preferable to more rigid top-down structures. However, effective engagement of multiple stakeholders can often be hindered by power imbalances (Gaventa 2006; Reed 2008; Siangulube et al. forthcoming), the influence of which can be exacerbated when attempting engagement across governance levels (Gallemore et al. 2015). Therefore, in practice, the increased integration of state and non-state actors across different levels must be carefully implemented, with a consideration of power asymmetries and existing access and tenure rights. Conducting a participatory theory of change exercise can help in this regard by building trust, enhancing transparency, and acting as a useful pre-cursor to establishing actors’ roles and responsibilities.

Participants suggested that landscape governance can be improved through *enhanced awareness, education, and training* for sustainable natural resource management, acknowledging socio-cultural and environmental dimensions. While community-based training programmes were specifically encouraged by participants, it was recognized that awareness-raising and knowledge production should be participatory processes that engage multiple knowledge holders and can therefore reflect, and incorporate, plural values. They considered that such processes could help to clarify both the means and benefits of sustainable natural resource management. Rigorously designed and implemented participatory monitoring and citizen science approaches can further enhance inclusivity, data transparency, and accessibility. Finally, participants recommended the need for greater *clarity and enforcement of rules and laws*, with particular emphasis on defining chiefdom boundaries, harmonization of customary and statutory laws, and clear rules on encroachment and access to natural resources.

### Policy considerations

Across sectors and scales participants identified a need to strengthen governance capacity and to develop strategies that enhance the clarity of, and access to, policy initiatives. There was a demand for improved access to land-use maps, impending land acquisitions or annexing and improved dialogue concerning land and natural resource-use rights and boundaries, particularly those demarcating chiefdoms and reserves. Finally, there was a call for greater support for, and expansion of, extension services (e.g. knowledge, inputs, and credits) for sustainable agriculture and natural resource management.

Recent policy development in Zambia has demonstrated a commitment towards economic diversification and decentralized governance via multi-sectorial integration, indicating a favorable enabling environment for integrated landscape approaches (O’Connor et al. 2021). However, participants observed that oftentimes there is a conflict between policies formulated at the national level and local needs, capacity, and objectives at the landscape level (see also Reed et al. 2015a; Chia and Sufo 2016). Furthermore, there is a tendency for actors to engage with ‘like-minds’, that is those operating in the same sector and/or within the same governance level – whether that be national, district, or local (Di Gregorio et al. 2019). Such institutional fragmentation serves to undermine policy performance (Brockhaus and Angelsen 2012). Therefore, based on this exercise we provide the following suggestions for policy consideration.

Single sector environmental or agricultural policy models that disregard local socio-economic and cultural needs and aspirations are likely to fail or be unsustainable. Rather, smart policy mixes are required to address complex social-ecological challenges adequately and equitably (Law et al. 2016; Carmenta et al. 2020). Recent momentum suggests an opportunity for policy development at the national level to be increasingly integrated utilizing and enhancing current mechanisms that promote joined-up interventions from across policy domains. For example, commitments to cross-sectoral planning in both the current national development plan (7NDP) and biodiversity strategy and action plan (NBSAP2) show promise but likely must be further negotiated at sub-national levels to be successfully realized (O’Connor et al. 2021).

Policy design can be improved by increased horizontal integration as suggested above, but the way policies evolve and ultimately perform are influenced by place and multi-level governance contexts. Co-developed theory of change models can contribute towards evidence-based policy development by revealing place-based challenges of common concern and proposing locally relevant solutions. Meanwhile, increasing the interaction of political actors and landscape stakeholders can further help to overcome sector and scale silos and identify synergies between local aspirations and pledges made at the national scale towards global commitments (Adger et al. 2005; Cash et al. 2006).

Utilizing boundary organizations that facilitate dialogue between experts, decision-makers, and practitioners can support knowledge and policy production. The Zambia Community Based Natural Resource Management Forum (ZCBNRMF) is an example of one such organization that provides a platform for CBNRM discourse and development and has a strong reputation for partnership building, influencing policy and supporting communities. Our results suggest a well-designed theory of change building exercise can fundamentally support such endeavors by revealing which actors should be responsible for which roles and which actor coalitions need to be established, strengthened, or even confronted, to move towards more sustainable, and equitable, landscape management. Further, the development—or maintenance and strengthening—of shared learning platforms or multi-stakeholder forums can enhance collective learning and action, inform evidence-based and locally relevant policy-making, and foster behavioral change through policy interventions (Ros-Tonen et al. 2018; Barletti et al. 2020).

### Opportunities and challenges for theory of change and integrated landscape approaches

Environmental problems are complex, cross-sectorial, and affect different actors in different ways (Game et al. 2014). As such, the development of cross-sector partnerships such as integrated landscape approaches can help to bridge gaps between practitioners and policymakers, aid negotiation across different levels and develop integrated solutions to clearly interconnected problems related to food, water, climate, and livelihoods. However, effective implementation and sustainability of integrated landscape management is hindered by issues such as poor engagement, entrenched power relations, conflicting stakeholder visions, and lack of human and financial capacity, amongst others (Reed et al. 2020a; b; Vermunt et al. 2020). We consider, and this experience has shown, that bringing stakeholders together to co-develop a theory of change can support implementation and identify measures to mitigate some of the described barriers and constraints. There are, of course, financial costs and logistical challenges associated with organizing such theory of change processes. However, implementing agents must also consider whether these costs outweigh the costs of inaction that could lead to interventions that are poorly designed, targeted, or received by local actors.

Adopting integrated landscape approaches should be regarded as a long-term process and involves thinking beyond project timelines—this alleviates constraints associated with short-term targets, but incurs challenges for identifying medium-term indicators and specific end points and outcome objectives. Similarly, developing a theory of change—particularly for a social–ecological system—also presents challenges of where to conclude causal pathways (Qiu et al. 2018)—should the final box (goal/objective) in the theory of change be a social or environmental outcome? Given that participants will represent different epistemological backgrounds, achieving consensus on the overall objective could be a challenge and might require developing multiple causal pathways before attempting a synthesis. Further, integrated landscape approaches—and theories of change—are not a single intervention, but more typically a collection of activities, interventions, and deliberations. This participatory theory of change exercise suggested that integrated landscape approaches in Kalomo have the potential to be a long-lasting change vehicle and it was encouraging to see signs of consensus among stakeholders with differing values, needs, and objectives.

Nevertheless, while this exercise encouraged participants to reflect on past events and develop a shared vision of a desired future, the output inevitably represents a snapshot in time. However, the theory of change must not remain a snapshot—there is a need for dissemination and rigorous annual reflection, a need to revisit indicators, check progress, and ensure indicators and objectives remain appropriate, desirable, and contribute towards enhancing system resilience. Furthermore, issues of rights and equitable participation should remain central and, in line with ILA principles, there should be a commitment to pursue multi-level and cross-sectoral collaboration. Essentially, there needs to be an ongoing process of negotiation to help determine whether the landscape is moving towards the desired state and identify who is winning and who is losing. This requires a functional governance system in place that can learn from results, experiences, and mistakes to reflect and make necessary corrections or interventions that reorient to improve management plans and practices. Recent research developments can help here, for example, using governance evaluation frameworks that enable stakeholders to assess the status of governance and identify options for improvement (Kusters et al. 2020).

Temporality and issues of scale are therefore important factors to consider when attempting to improve landscape governance (Cash et al. 2006). Using the process of developing a theory of change to have participants negotiate and distinguish short-, medium-, and long-term goals is useful and helps to highlight potential trade-offs and synergies by making potentially contrasting, or even conflicting, objectives visible to all. This also ensures greater clarity of goals and the required stakeholder roles and responsibilities to pursue them. In doing so, identification of necessary policy changes or system interventions can also help to identify capacity development needs, and these needs can be based on previously outlined common concerns. To understand contextual cross-scale dynamics, research again has an important role to play and analyses of the ‘politics of scale’ can reveal how power relations and actor coalitions manifest across levels of governance (Young 2002; Görg 2007; Reed et al. 2020a, b).

Ensuring concerns are indeed common requires also ensuring that there is adequate representation of actors. A limitation of the exercise described here was the limited engagement of both local community members (below village head person status) and private sector actors. The latter were repeatedly invited, but failed to attend. It is known that participatory stakeholder engagement involves high transaction costs (Reed 2008). This is particularly problematic for poorly resourced groups and actors who may consider participation expensive in time or financial terms as they have to trade off against losing a day’s labour, but also for high-income actors who may fail to see the benefit of participating or consider that their participation could result in financial or other resource losses. Either way, high transaction costs can potentially reinforce existing power dynamics and inequalities (Gallemore et al. 2015). Investment and careful facilitation are therefore required to ensure fair representation. A triangulated research approach using established, complementary methods such as social network analysis and stakeholder perceptions (Reed et al. 2020a; b) along with an appropriate evaluation strategy (Chervier et al. 2020) can help to validate objectives and actions developed within multi-stakeholder discussions as part of integrated landscape approaches.

To enhance the potential of integrated landscape approaches to improve local well-being, enhance landscape resilience, and make resource use sustainable, bridging sectoral and scale gaps is crucial. We consider developing a participatory theory of change to be an important component of integrated landscape approach implementation, as it clearly describes the causal links required to move from intervention, through intermediary outcomes and to a desired future state. Doing so also helps to build trust amongst diverse stakeholders and encourage local buy-in, makes objectives and assumptions explicit, helps to clarify roles and responsibilities, and can support monitoring and evaluation, irrespective of which evaluation approach is selected (Chervier et al. 2020). Finally, despite the need for contextualization in applying landscape approaches, we feel there is high potential for extrapolating theory of change methods to other contexts, whether that be to other locations or to address other social–ecological sustainability challenges.

### Study limitations

As might be expected from a process that attempts to match multiple stakeholder objectives with a high degree of complexity, our study suffered from a number of limitations. Firstly, we had several challenges related to stakeholder engagement. Despite our best attempts to engage private sector actors in the process, there were regrettably no participants from this sector. Rather than speculating why they neglected to engage, we have subsequently designed a follow-up study that will focus singularly on private sector actors in Kalomo within the maize, livestock, tobacco, and charcoal industries. We hope that this will provide some guidance and ultimately lead to greater engagement of these actors within multi-stakeholder dialogue processes in the region. We also felt that there was limited representation of local community members and farmers, despite the presence of a number of village head people. Finding the right balance between adequate representation and a manageable number of participants will always be a challenge. We intended to address this issue by conducting community consultations post-workshop, but this was constrained by other factors beyond our control (see below). This remains an activity which we intend to deliver when possible. Finally, due to time and resource constraints we were unable to secure independent facilitation for the workshop. As mentioned, the facilitators were adequately experienced and trained, but ideally (and in our future processes) fully independent facilitation would be used.

When planning the participatory theory of change workshop, we had allocated time post-workshop for a full debrief, to conduct one on one interviews, and to begin the process of community consultation (step 8 in Fig. 4). However, in the days immediately following the workshop, national civil unrest emerged due to a series of chemical gas attacks on members of the public and subsequent retaliatory mob violence.^2^ This then led to a military-led lockdown that meant our planned activities were inevitably postponed. The global COVID-19 pandemic then further delayed these plans, which meant that we were still unable to conduct steps 8 and 9 (see Fig. 4) of the participatory theory of change process. These steps are hugely important, as they enable a broader consultation process that can help ensure that the proposed theory of change is locally relevant and demanded. These steps also guide the design of intervention (and monitoring) strategies and identify specific roles and responsibilities—crucial in ensuring that the theory of change process is developed into a theory of change-based action plan for future management. As the situation improves in Zambia, we will resume these activities. In the meantime, we have conducted several virtual stakeholder workshops to refine the details of the theory of change synthetic and begin the process of establishing roles and responsibilities moving forward.

## Conclusion

Landscapes are complex, dynamic systems, subject to stochastic changes and influenced by the needs and demands of a wide range of stakeholders. Finding a balance between meeting globally conceived environmental targets, national production goals, and supporting local well-being is a persistent challenge in the Kalomo landscape and within sub-Saharan African landscapes more broadly. Integrated landscape approaches are examples of strategies that attempt to reconcile such competing claims to land through increased and improved stakeholder negotiation. Such approaches have become increasingly widespread with support from the policy, donor, and research domains. However, their effectiveness and optimal means of implementation remain inconclusive. What is clear is that implementers will need to employ a range of tools and methods to account for multiple objectives perhaps being pursued through multiple actions and interventions. The theory of change concept has been applied across a range of fields and has been promoted for use within integrated landscape approaches, but has thus far been lacking in application. Here, we have attempted to address this gap and recommend that the theory of change should be a key component of the toolbox available for integrated landscape approaches.

The theory of change concept emphasizes the value of incorporating multiple stakeholders, creating a shared vision, and demonstrating willingness to adapt (Rice et al. 2020). We show that engaging a diverse stakeholder group to co-develop a theory of change for landscape management can contribute towards helping build trust across previously distinct stakeholder groups by engaging in dialogue to establish commonly shared concerns and visions for the future. Furthermore, stakeholders were able to identify the necessary actions required to move towards more sustainable landscape management in Kalomo. We expect the theory of change output and planned future consultation processes have potential to drive more effective environmental policy development and performance through enhanced integration, both horizontally (engaging across ministries within the same level of government) and vertically (engaging in multi-level governance). Importantly, this process of a participatory theory of change not only encourages stakeholders to question what is wrong with the current system and envision how a different future system might look, but also to critically consider how a just and equitable transition from one to the other might transpire.

## Footnotes

1 https://www2.cifor.org/colands/.

2 https://www.youtube.com/watch?v=tdI5Bs0mFEg&ab_channel=Mark1333.

## References

[CR1] Abukari H, Mwalyosi R (2020). Local communities’ perceptions about the impact of protected areas on livelihoods and community development. Glob Ecol Conserv.

[CR2] Acheampong EO (2020). Application of landscape approach principles motivates forest fringe farmers to reforest Ghana’s degraded reserves. Forests.

[CR1001] Adger WN, Brown K, Tompkins EL (2005) The political economy of cross-scale networks in resource co-management. Ecol Soc 10(2)

[CR3] Barlow J (2018). The future of tropical hyperdiverse ecosystems. Nature.

[CR4] Bennett NJ (2016). Using perceptions as evidence to improve conservation and environmental management. Conserv Biol.

[CR5] Biggs D (2016). Developing a theory of change for a community-based response to illegal wildlife trade. Conserv Biol.

[CR6] Brandt J (2003). Multifunctional landscapes—perspectives for the future. J Environ Sci.

[CR7] Breuer E (2016). Using theory of change to design and evaluate public health interventions: a systematic review. Implement Sci.

[CR8] Brockhaus M, Angelsen A, Angelsen A (2012). Seeing REDD+ through 4Is: a political economy framework. Analysing REDD+: challenges and choices.

[CR9] Carmenta R (2017). Perceptions across scales of governance and the Indonesian peatland fires. Glob Environ Chang.

[CR10] Carmenta R (2020). Characterizing and evaluating integrated landscape initiatives. One Earth.

[CR11] Cash DW et al (2006) Scale and cross-scale dynamics: governance and information in a multilevel world. Ecol Soc 11(2)

[CR12] Chazdon RL (2021). Key challenges for governing forest and landscape restoration across different contexts. Land Use Policy.

[CR13] Chervier C, Piketty MG, Reed J, Reed J, Ros-Tonen MAF, Sunderland T (2020). Theories of change and monitoring and evaluation types for landscape approaches. Operationalizing integrated landscape approaches in the tropics.

[CR14] Chia EL, Sufo RK (2016). A situational analysis of Cameroon’s Technical Operation Units (TOUs) in the context of the landscape approach: critical issues and perspectives. Environ Dev Sustain.

[CR500] CSO (2012) 2010 Census of population and housing. Population summary report. March 2012. Central statistical office, Lusaka, Zambia

[CR15] Di Gregorio M (2019). Multi-level governance and power in climate change policy networks. Glob Environ Change.

[CR16] Díaz S (2019). Pervasive human-driven decline of life on Earth points to the need for transformative change. Science.

[CR17] Djenontin INS, Meadow AM (2018). The art of co-production of knowledge in environmental sciences and management: lessons from international practice. Environ Manag.

[CR18] Djenontin INS, Zulu LC (2021). The quest for context-relevant governance of agro-forest landscape restoration in Central Malawi: insights from local processes. For Policy Econ.

[CR19] Erbaugh JT, Agrawal A (2017). Clarifying the landscape approach: a letter to the editor on “Integrated landscape approaches to managing social and environmental issues in the tropics”. Glob Change Biol.

[CR20] Estrada-Carmona N (2014). Integrated landscape management for agriculture, rural livelihoods, and ecosystem conservation: an assessment of experience from Latin America and the Caribbean. Landsc Urban Plan.

[CR21] Freeman OE, Duguma LA, Minang PA (2015). Operationalizing the integrated landscape approach in practice. Ecol Soc.

[CR22] Gallemore C (2015). Transaction costs, power, and multi-level forest governance in Indonesia. Ecol Econ.

[CR23] Game ET (2014). Conservation in a wicked complex world; challenges and solutions. Conserv Lett.

[CR24] García-Martín M (2016). Integrated landscape initiatives in Europe: multi-sector collaboration in multi-functional landscapes. Land Use Policy.

[CR25] Gaventa J (2006). Finding the spaces for change: a power analysis. IDS Bull.

[CR26] Görg C (2007). Landscape governance the “politics of scale” and the “natural” conditions of places. Geoforum.

[CR27] Hoffmann S, Pohl C, Hering JG (2017). Methods and procedures of transdisciplinary knowledge integration: empirical insights from four thematic synthesis processes. Ecol Soc.

[CR28] Kusters K (2020). Inclusive landscape governance for sustainable development: assessment methodology and lessons for civil society organizations. Land.

[CR1002] Law EA (2016). Mixed policies give more options in multifunctional tropical forest landscapes Mixedpolicies give more options in multifunctional tropical forest landscapes. J Appl Ecol.

[CR29] Mbanga TM, Mulenga MC, Membele G (2021). Monitoring forest cover change in Kalomo Hills local forest using remote sensing and GIS: 1984–2018. J Remote Sens GIS.

[CR30] Mcginnis MD (2016) Polycentric governance in theory and practice: dimensions of aspiration and practical limitations. Available at SSRN 3812455

[CR31] McShane TO (2011). Hard choices: making trade-offs between biodiversity conservation and human well-being. Biol Conserv.

[CR32] Meinig DW, Meinig DW (1979). The beholding eye. Ten versions of the same scene. The interpretation of ordinary landscapes.

[CR33] Milder JC (2014). Integrated landscape initiatives for African agriculture, development, and conservation: a region-wide assessment. World Dev.

[CR34] Moombe KB, Reed J, Ros-Tonen M, Sunderland T (2020). Understanding landscape dynamics: a case study from Kalomo District. Operationalizing integrated landscape approaches in the tropics.

[CR35] Nagendra H, Ostrom E (2012). Polycentric governance of multifunctional forested landscapes. Int J Commons.

[CR36] Narayanasamy N (2009). Participatory rural appraisal: principles, methods and application.

[CR37] Nchito WS (2014). The growth and functions of small urban centres in Zambia: a case study of Mazabuka and Kalomo.

[CR38] Newig J, Koontz TM (2014). Multi-level governance, policy implementation and participation: the EU’s mandated participatory planning approach to implementing environmental policy. J Eur Publ Policy.

[CR39] Norström AV (2020). Principles for knowledge co-production in sustainability research. Nat Sustain.

[CR40] O’Connor A, Gumbo D, Moombe KB (2021) Potential for integrated landscape approaches: a review of Zambia’s national environment and development policies. CIFOR InfoBrief, Bogor, Indonesia. 10.17528/cifor/007954

[CR41] Omoding J (2020). Implementing a landscape approach in the Agoro-Agu region of Uganda. Parks.

[CR42] Pohl C (2021). Conceptualising transdisciplinary integration as a multidimensional interactive process. Environ Sci Policy.

[CR43] Qiu J (2018). Evidence-based causal chains for linking health, development, and conservation actions. Bioscience.

[CR44] Rasmussen LV (2017). Bridging the practitioner-researcher divide: indicators to track environmental, economic, and sociocultural sustainability of agricultural commodity production. Glob Environ Change.

[CR45] Reed MS (2008). Stakeholder participation for environmental management: a literature review. Biol Conserv.

[CR46] Reed J, Deakin L, Sunderland T (2015). What are “Integrated Landscape Approaches” and how effectively have they been implemented in the tropics: a systematic map protocol. Environ Evid.

[CR47] Reed J, van Vianen J, Sunderland T (2015b) From global complexity to local reality: aligning implementation pathways for the Sustainable Development Goals and landscape approaches. CIFOR InfoBrief (Vol. 129), Bogor, Indonesia. 10.17528/cifor/005865

[CR48] Reed J (2016). Integrated landscape approaches to managing social and environmental issues in the tropics: learning from the past to guide the future. Glob Change Biol.

[CR49] Reed J (2017). Have integrated landscape approaches reconciled societal and environmental issues in the tropics?. Land Use Policy.

[CR50] Reed J, Reed J, Ros-Tonen MAF, Sunderland T (2020). A methods toolbox for integrated landscape approaches. Operationalizing integrated landscape approaches in the tropics.

[CR51] Reed J (2020). Integrated landscape approaches in the tropics: a brief stock-take. Land Use Policy.

[CR900] Reed J, Ros-Tonen MAF, Sunderland TCH (2020c) Operationalizing integrated landscape approaches in the tropics. CIFOR

[CR52] Rice WS, Sowman MR, Bavinck M (2020). Using theory of change to improve post-2020 conservation: a proposed framework and recommendations for use. Conserv Sci Pract.

[CR53] Ros-Tonen MAF, Reed J, Sunderland T (2018). From synergy to complexity: the trend toward integrated value chain and landscape governance. Environ Manag.

[CR1003] Barletti JPS (2020). Designing for engagement: a realist synthesis review of how context affectsthe outcomes of multi-stakeholder forums on land use and/or land-use change. World Deve.

[CR54] Sayer JA, Wells MP, McShane TO, Wells MP (2004). The pathology of projects. Getting biodiversity projects to work.

[CR55] Sayer J (2013). Ten principles for a landscape approach to reconciling agriculture, conservation, and other competing land uses. Proc Natl Acad Sci USA.

[CR56] Sayer J (2014). Landscape approaches; what are the pre-conditions for success?. Sustain Sci.

[CR57] Sayer JA (2016). Measuring the effectiveness of landscape approaches to conservation and development. Sustain Sci.

[CR58] Scherr SJ, Shames S, Friedman R (2013) Defining integrated landscape management for policy makers, EcoAgriculture policy focus, vol 10, pp 1–6

[CR59] Stafford-smith M (2017). Integration: the key to implementing the Sustainable Development Goals. Sustain Sci.

[CR60] Sunderland T (2009). Bridging the gap: how can information access and exchange between conservation biologists and field practitioners be improved for better conservation outcomes?. Biotropica.

[CR61] Tengö M (2014). Connecting diverse knowledge systems for enhanced ecosystem governance: the multiple evidence base approach. Ambio.

[CR62] Toomey AH et al (2015) Inter- and trans-disciplinary research: a critical perspective. GSDR Brief, pp 1–3

[CR63] Tress B (2001). Bridging human and natural sciences in landscape research. Landsc Urban Plan.

[CR64] Tscharntke T (2012). Global food security, biodiversity conservation and the future of agricultural intensification. Biol Conserv.

[CR65] USAID LESTARI (2019) Lessons learned technical brief. Jakarta

[CR66] Vermunt DA, Verweij PA, Verburg RW (2020). What hampers implementation of integrated landscape approaches in rural landscapes?. Curr Landsc Ecol Rep.

[CR67] Vogel I (2012) Review of the use of ‘Theory of Change’ in international development. Department for International Development (DFID), UK, vol 10

[CR68] Young OR (2002) Institutional interplay: the environmental consequences of cross-scale interactions. In: Ostrom E et al (eds) The drama of the commons. National Academy Press, pp 263–291

[CR69] Zanzanaini C (2017). Integrated landscape initiatives for agriculture, livelihoods and ecosystem conservation: an assessment of experiences from South and Southeast Asia. Landsc Urban Plan.

